# A case of incarcerated gravid uterus with a history of cesarean section was a good candidate for manual reduction: A case report

**DOI:** 10.1002/ccr3.3524

**Published:** 2020-11-12

**Authors:** Sayaka Suzuki, Soichiro Obata, Mariko Utsunomiya, Etsuko Miyagi, Shigeru Aoki

**Affiliations:** ^1^ Perinatal Center for Maternity and Neonates Yokohama City University Medical Center Yokohama Japan; ^2^ Department of Obstetrics and Gynecology Yokohama City University Hospital Yokohama Japan

**Keywords:** cesarean section, myoma, pregnancy

## Abstract

Because the anterior lower uterine segment is stretched, the incarcerated gravid uterus case with a history of cesarean section is a good candidate for manual reduction.

## Graphical abstract

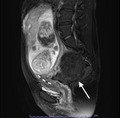

## 1 INTRODUCTION

An incarcerated gravid uterus is a condition wherein the gravid uterus is retroverted and the uterine fundus is located in Douglas' pouch. This is caused by conditions such as uterine fibroids or endometriosis, which abnormally stretch the uterine cervix and the anterior lower uterine segment.^1^, ^2^ Occasionally, the uterus is retroverted in early pregnancy, which is spontaneously reversed at 14‐16 weeks of gestation.^2^ The retroversion rarely persists beyond 16 weeks of gestation, and only one in 3000‐10 000 cases remains so until delivery.^1^, ^2^, ^3^ The incarcerated gravid uterus is a risk factor for fetal growth restriction (FGR), miscarriage, premature delivery, and uterine rupture.^1^ The stretched anterior lower uterine segment is at a higher risk of uterine rupture, especially in pregnancies following previous cesarean sections.^1^, ^2^

We report a case of incarcerated gravid uterus with a history of previous cesarean section, wherein we successfully performed manual reduction at 20 weeks of gestation. Written informed consent was obtained from the patient for the publication of this case report.

## 2 CASE

A 37‐year‐old woman, gravida 3, para 1, presented to us with a history of cesarean section for the incarcerated gravid uterus 3 years prior. During her previous pregnancy, an 8‐cm uterine fibroid was identified in Douglas' pouch, and the incarcerated gravid uterus was diagnosed. Consequently, a cesarean section was performed at 37 weeks and 5 days of gestation. A midline laparotomy was performed from above the umbilical ring to 2 cm above the pubis, followed by reduction in the incarcerated gravid uterus. The neonate was subsequently delivered through an anterior lower uterine segment incision.

**FIGURE 1:**
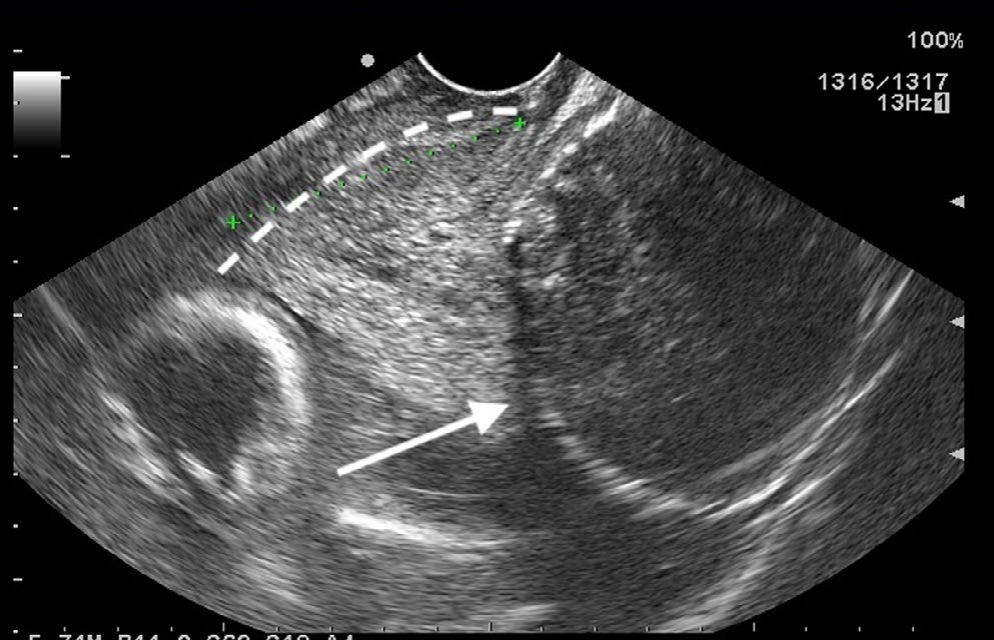
Transvaginal ultrasonography at 17 wk of gestation. The cervix (dotted white line) is displaced forward, and an 8‐cm uterine fibroid (white arrow) is visible in Douglas' pouch

**FIGURE 2:**
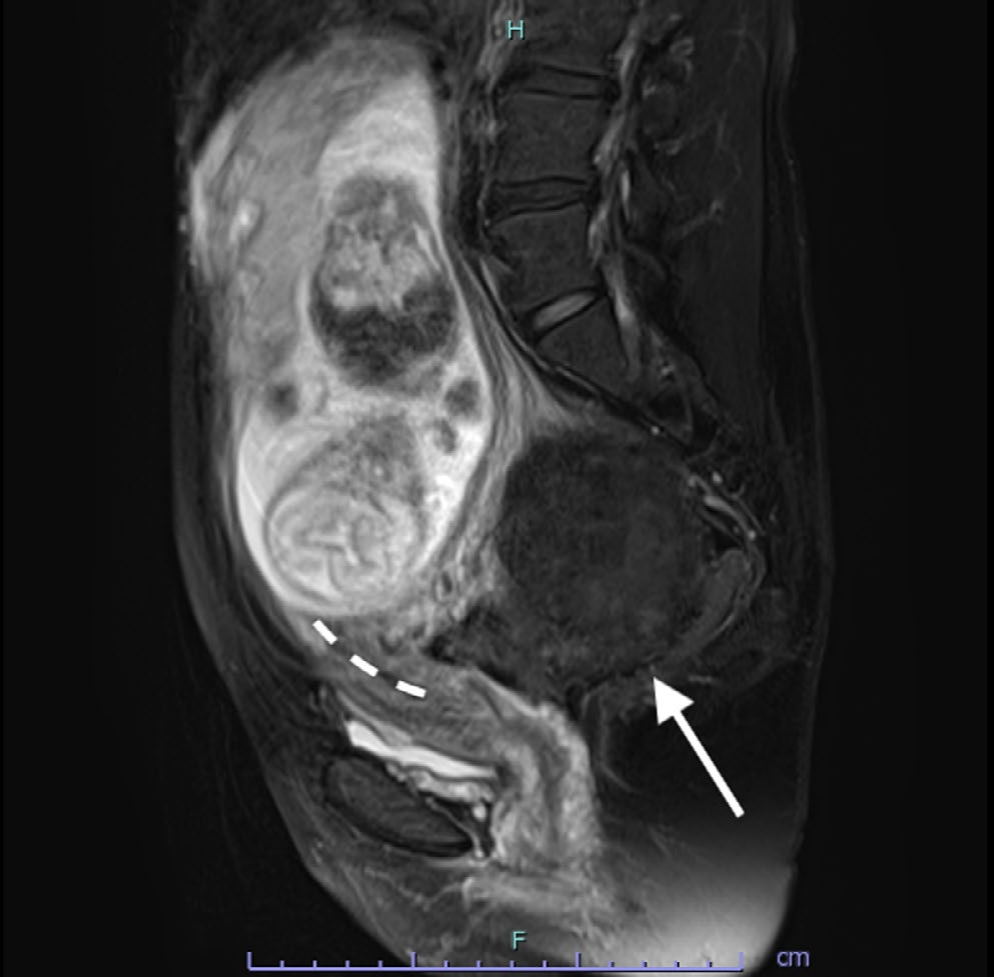
Magnetic resonance image at 19 wk of gestation (sagittal, T2‐weighted). The image shows an excessively stretched uterine cervix, one of the characteristic symptoms of the incarcerated gravid uterus. The uterus appears severely retroverted, and the uterine fibroid (white arrow) is visible in Douglas' pouch

The present spontaneous pregnancy occurred 3 years following the previous cesarean section. The patient consulted our outpatient clinic at 5 weeks of gestation. Initial imaging confirmed that the 8‐cm uterine fibroid was still located in Douglas' pouch. At 17 weeks of gestation, transvaginal ultrasonography identified a uterine fibroid in Douglas' pouch, with significant stretching of the uterine cervix, suggesting a diagnosis of incarcerated gravid uterus (Figure 1), which was confirmed by magnetic resonance imaging (MRI) (Figure 2). In view of her previous history of persistent incarcerated gravid uterus that necessitated cesarean section, a spontaneous reduction was not expected and the patient was defined as having a higher risk of uterine rupture, and we consequently decided to perform manual reduction in the incarcerated gravid uterus.

**FIGURE 3:**
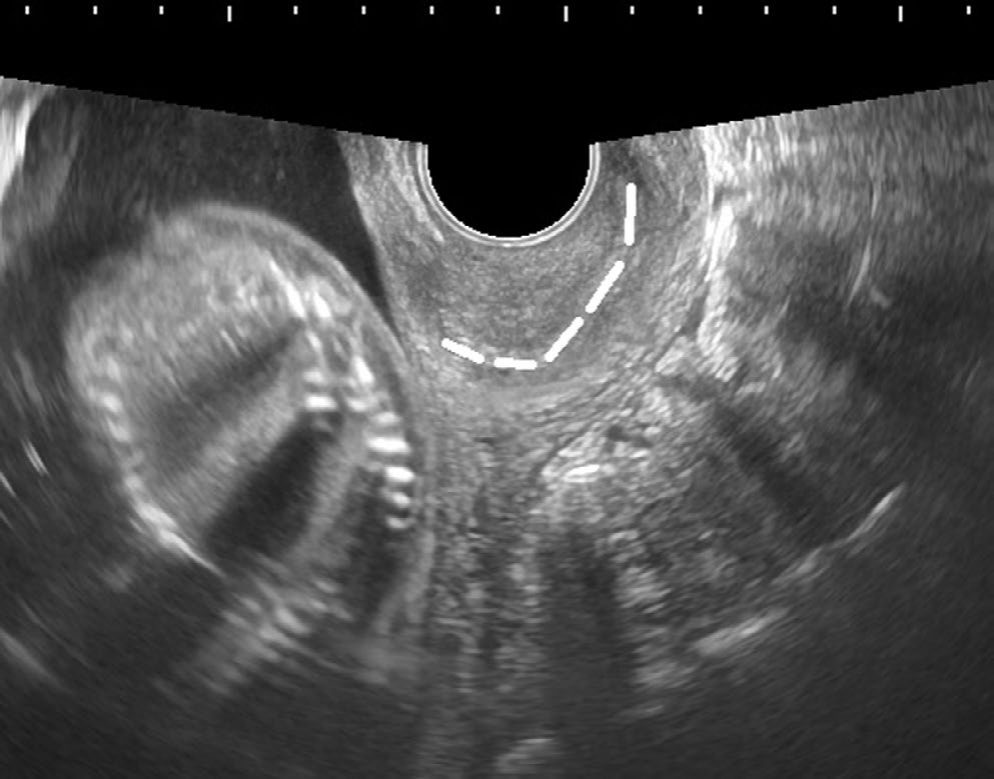
Transvaginal ultrasonography at 20 wk of gestation (after manual reduction). The cervix (dotted white line) is positioned normally and is not stretched, contrary to its previous appearance

**FIGURE 4:**
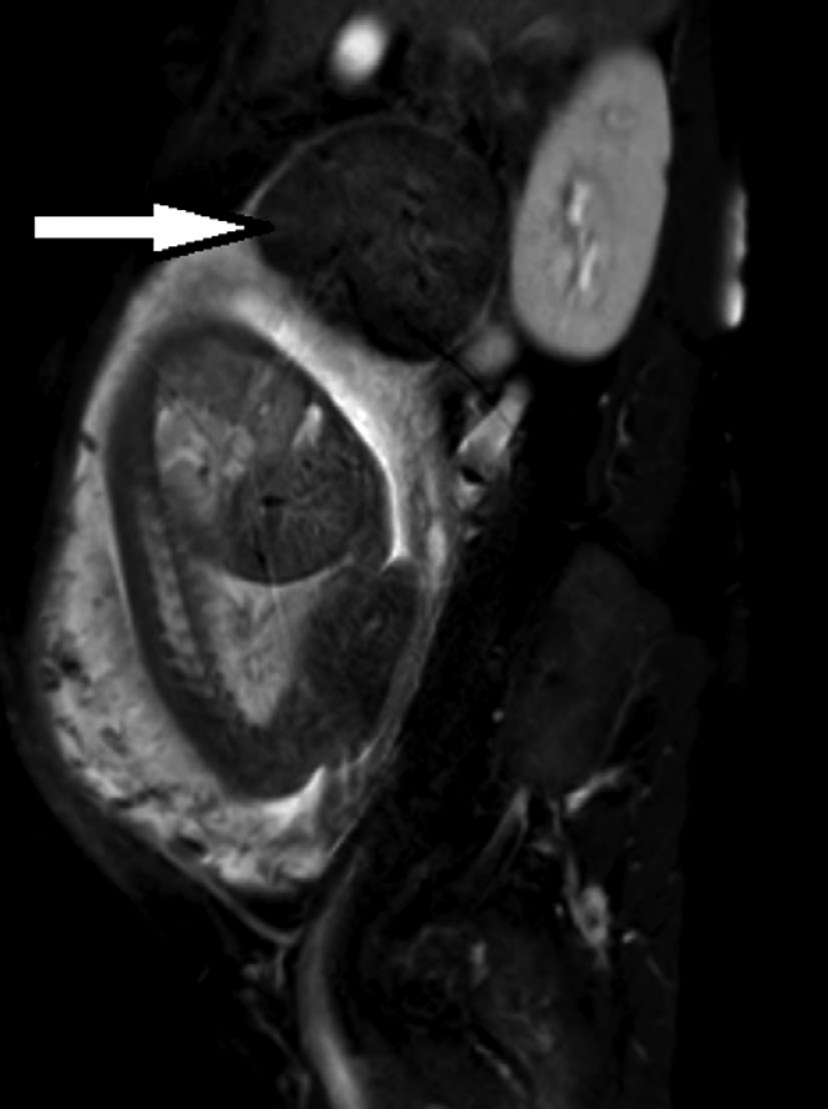
Magnetic resonance image at 34 wk of gestation (sagittal, T2‐weighted). The uterine fibroid (white arrow) is located on the maternal cranial side

**FIGURE 5:**
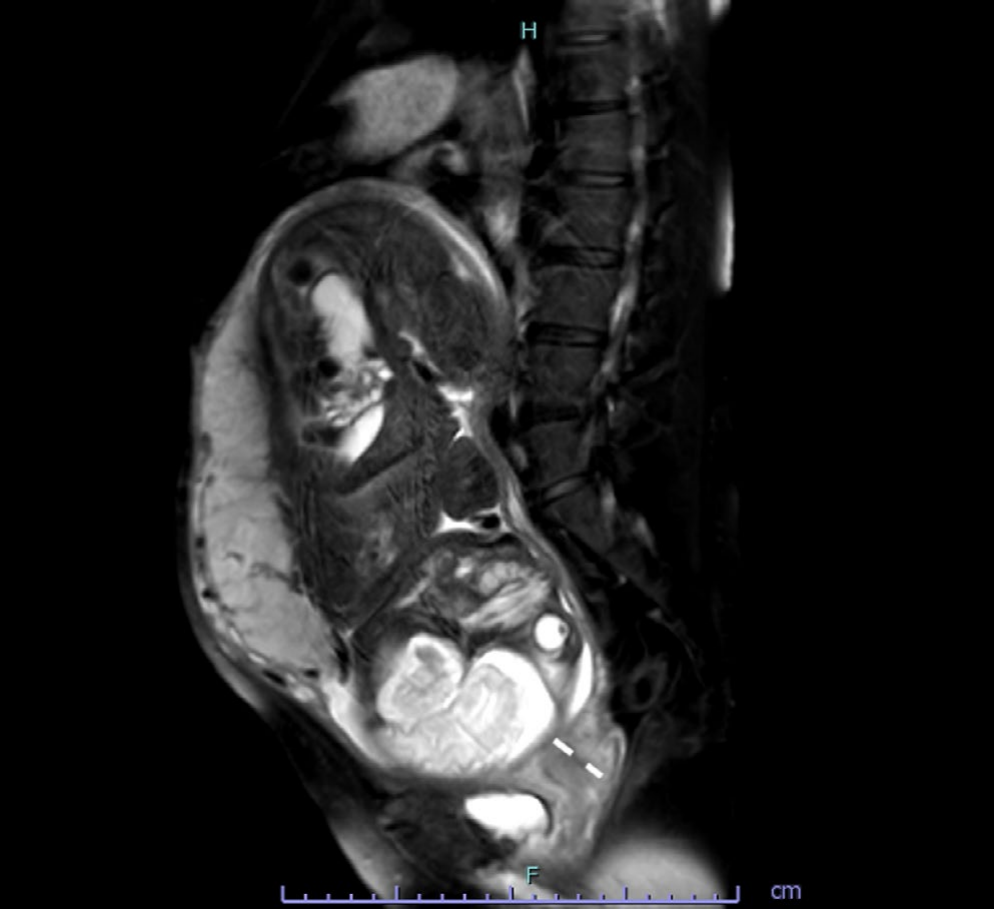
Magnetic resonance image at 34 wk of gestation (sagittal, T2‐weighted). The uterine cervix is positioned normally, and the excessively stretched uterine cervix is resolved (dotted white line)

At 20 weeks and 1 day of gestation, we performed manual reduction in the uterus under spinal anesthesia. We raised the uterus by a transabdominal approach, and concurrently pushed the uterine fibroid vaginally, to achieve successful reduction. After the procedure, the position of the uterine cervix was normalized, and there was a visible improvement in the stretch of the uterine cervix and anterior lower uterine segment (Figure 3). Thereafter, we followed her up at the outpatient clinic as there were no signs of uterine rupture, threatened preterm labor, or FGR. MRI performed at 34 weeks of gestation identified that the uterine fibroid was located at the maternal cranial side, and not in Douglas' pouch, confirming the successful reduction in the incarcerated gravid uterus (Figures 4 and 5). When we performed cesarean section at 38 weeks of gestation, there was no evidence of any thinning of the uterine myometrium suggestive of uterine rupture. The male neonate had a birthweight of 3200 g, and Apgar scores of 8 and 9 at 1 and 5 minutes, respectively. To prevent further recurrence of the incarcerated gravid uterus in subsequent pregnancies, we concurrently performed fibroid enucleation. The tumor was diagnosed as a uterine fibroid by histopathological analysis. The patient did not have any postoperative complications and was discharged on postoperative day 6.

We obtained her consent to publish this study as a case report.

## 3 DISCUSSION

In case of an incarcerated gravid uterus, the uterine cervix and the anterior lower uterine segment are excessively stretched (Figure 2). This is a risk factor for uterine rupture, especially in cases with a history of a cesarean section in particular. The reduction in the incarcerated gravid uterus not only improves the stretching of the uterine cervix and the anterior lower uterine segment (Figure 5) but also reduces the risk of uterine rupture.

Recurrence of incarcerated gravid uterus may be prevented by correcting its risk factors. Several cases of recurrent incarcerated gravid uterus have been reported.^4^, ^5^, ^6^, ^7^, ^8^ In most of these cases, no obvious cause or risk factors were identified. In our case, there was a uterine fibroid trapped in Douglas' pouch that appeared to contribute to the incarceration of the gravid uterus. We did not perform fibroid enucleation during the previous cesarean section for two reasons: First, this uterine fibroid was asymptomatic prior to her pregnancy; second, this procedure could cause a higher risk of bleeding during cesarean section.^9^ However, there are previous reports wherein fibroid enucleation was performed concurrently with cesarean section, with well‐trained operators using appropriate techniques.^10^, ^11^ In this case, our patient was planning a third pregnancy; therefore, we perform fibroid enucleation concurrent with cesarean section.

All cases that undergo cesarean section are at a higher risk of uterine rupture during their next pregnancy.^12^ This is especially true in the case of incarcerated gravid uterus, where the risk with cesarean section is compounded by excessive stretching of the anterior uterine lower segment.^1^, ^2^ Therefore, our case appeared to be a good candidate for manual reduction to prevent this complication, as well as for improving other pregnancy outcomes. Similar successful reductions followed by delivery at term have been reported.^5^, ^7^ Moreover, in one case without reduction, increased uterine contractions and delivery by cesarean section at 34 weeks of gestation have been reported.^6^ Considering the previous obstetric history in our case, the possibility of spontaneous reduction in the incarcerated uterus seemed to be extremely low. Therefore, the successful manual reduction performed in our case may be considered an important decision that contributed to the safe completion of her pregnancy.

In general, a retroverted uterus in early pregnancy will reduce spontaneously by 16 weeks of gestation.^2^ The possibility of spontaneous reduction after 16 weeks of gestation is low and should prompt consideration of active reduction. In some cases, reduction may be obtained by urination followed by the chest‐knee position. If this maneuver is unsuccessful, a manual reduction under anesthesia should be attempted. It is important to recognize the possibility of adhesions in cases with history of previous laparotomy. The complications of manual reduction include placental abruption, preterm delivery, and intrauterine fetal death.^13^ There are case reports of reduction achieved with colonoscopy,^14^ laparoscopy, or laparotomy.^14^ These methods may be considered in the event of a failure of manual reduction. Reduction is considered to be difficult after 20 weeks of gestation.^2^, ^14^ Therefore, cases with failed reduction or late diagnosis should be managed carefully until a cesarean section is performed.^1^, ^2^ In this case, we diagnosed the case as an incarcerated uterus at 17 weeks of gestation and recommended manual reduction. However, it took time to obtain the patient's consent to perform the manual reduction. Therefore, we performed manual reduction at 20 weeks of gestation.

Our case report highlights the importance of accurate diagnosis and considers the timely intervention based on the patient's prior medical history in managing incarcerated gravid uterus to achieve favorable pregnancy outcomes. The accurate management of incarcerated gravid uterus warrants further investigation through an advanced multi‐institutional survey.

## CONFLICT OF INTEREST

None declared.

## AUTHOR CONTRIBUTIONS

SS: Contributed to the finalization of the manuscript and performed the surgery. SO: Contributed to the first draft and finalization of the manuscript. MU: Performed the surgery. EM and SA: Supervised the case report.

## ETHICAL APPROVAL

Prior to submission, appropriate consent for publication of images and data has been obtained.

## Data Availability

Data sharing not applicable to this article as no datasets were generated.
